# Identification of potential crucial genes in atrial fibrillation: a bioinformatic analysis

**DOI:** 10.1186/s12920-020-00754-5

**Published:** 2020-07-18

**Authors:** Junguo Zhang, Xin Huang, Xiaojie Wang, Yanhui Gao, Li Liu, Ziyi Li, Xuejiao Chen, Jie Zeng, Zebing Ye, Guowei Li

**Affiliations:** 1 Center for Clinical Epidemiology and Methodology (CCEM), Guangdong Second Provincial General Hospital, Guangzhou, 510317 Guangdong China; 2 School of Public Health, Guangdong Pharmaceutical University, Guangzhou, 510317 Guangdong China; 3 Department of Cardiology, Guangdong Second Provincial General Hospital, Guangzhou, 510317 Guangdong China; 4 Department of Health research methods, Evidence, and Impact (HEI), McMaster University, 1280 Main St West, Hamilton, ON L8S 4L8 Canada

**Keywords:** Potential crucial genes, Atrial fibrillation, Bioinformatic gene analysis, Gene expression omnibus, Biomarkers

## Abstract

**Background:**

Atrial fibrillation (AF) is at least partially heritable, affecting 2–3% of the population in Europe and the USA. However, a substantial proportion of heritability is still lacking. In the present study, we aim to identify potential crucial genes associated with AF through bioinformatic analyses of public datasets.

**Methods:**

Microarray data sets of GSE115574, GSE31821, GSE79768, GSE41177 and GSE14975 from the Gene Expression Omnibus (GEO) database were retrieved. After merging all microarray data and adjusting batch effect, differentially expressed genes (DEGs) were identified. Functional enrichment analyses based on Gene Ontology (GO) resource, Kyoto Encyclopedia of Genes and Genomes (KEGG) resource, Gene Set Enrichment Analysis (GSEA), Reactome Pathway Database and Disease Ontology (DO) were carried out. Protein-protein interaction (PPI) network was constructed using the STRING database. Combined with aforementioned significant bioinformatics information, potential crucial genes were subsequently selected. The comparative toxicogenomics database (CTD) was used to explore the interaction between potential crucial genes and AF.

**Result:**

We identified 27 of DEGs with gene expression fold change (FC) ≥ 1.5 or ≤ 2/3 (|log2 FC| ≥ 0.58) and 5 with FC ≥ 2 or ≤ 0.5 (|log2 FC| ≥ 1) of AF patients compared with sinus rhythm controls. The most significantly enriched pathway was regulation of insulin-like growth factor transport and uptake by insulin-like growth factor binding proteins. *IGFBP2, C1orf105, FHL2, CHGB, ATP1B4, IGFBP3, SLC26A9, CXCR4* and *HTR2B* were considered the potential crucial genes. CTD showed *CXCR4, IGFBP2, IGFBP3* and *FHL2* had higher scores with AF.

**Conclusions:**

The 9 potential crucial genes, especially *CXCR4, IGFBP2, IGFBP3* and *FHL2*, may be associated with risk of AF. Our study provided new insights of AF into genetics, molecular pathogenesis and new therapeutic targets.

## Background

Atrial fibrillation (AF) is the most common sustained arrhythmia and is one of the major causes of stroke, heart failure, sudden death, and cardiovascular morbidity in the world [1]. The estimated number of AF patients is 34 million worldwide, and gradually increasing with the aging of the population [2]. However, the pathophysiologic mechanism underlying many AF cases remains unclear, resulting in a lack of effective treatment [3]. Only a small number of AF patients can normalize heart rhythm by catheter ablation or cardiac surgery [4]. The higher prevalence and limited treatments of AF lead to substantial public health and economic burdens [5]. Therefore, it is necessary to improve our understanding of AF pathogenesis and to develop better screening methods for AF.

Epidemiological evidence over the last decade has identified that metabolic syndrome, pre-hypertension, obesity, obstructive sleep apnea, exercise, and dietary intake of stimulants are major risk factors for AF [4, 6]. However, only a small proportion of exposed individuals eventually developed AF, suggesting that a strong genetic component might be a risk factor contributing to the susceptibility of AF. From 2007 to 2017, genetic research in European, Asian, and African-American ancestry groups have reported 17 independent signals at 14 genomic regions, such as *PITX2*, *ZFHX3*, and *PRRX1*, associated with AF [7, 8]. In 2018, a preliminary genome-wide association study meta-analysis including over 93,000 AF cases and more than 1 million referents identified at least 134 genetic loci significantly associated with risk of AF [9]. However, combined with the results of the genetic studies thus far, genetic variation only accounts for 42% of the heritability for AF [9]. Notably, a substantial proportion of heritability is still lacking. One potential interpretation is that unidentified genes may partially contribute to the missing heritability. Therefore, there are still many related genes to be identified, which will help us better understand the pathogenesis of AF and facilitate the discovery of novel diagnostic biomarkers or therapeutic target.

In this study, we aimed to identify the potential crucial genes for AF through Gene Expression Omnibus (GEO) database using bioinformatic methods and to analyze their expression, function and interaction.

## Methods

### Atrial fibrillation datasets

Raw files of 5 registered microarray data sets, including GSE115574, GSE31821, GSE79768, GSE41177 and GSE14975 (Table 1), were downloaded from the NCBI GEO database (https://www.ncbi.nlm.nih.gov/geo/). All of these datasets were obtained from the microarray platform of Affymetrix Human Genome U133 Plus 2.0 Array [HG-U133_Plus_2]. In each data set, only human left atrial appendage (LAA) samples from AF and sinus rhythm (SR) subjects were selected, and finally 46 AF and 31 SR samples were included for subsequent analyses.

**Table 1:** Characteristics of datasets in this study

GSE series	Platform	Total	AF	SR	Country	Contributors
GSE115574	GPL570	29	14	15	Turkey	Deniz GC et.al
GSE31821	GPL570	6	4	2	France	Morel E et.al
GSE79768	GPL570	13	7	6	Taiwan	Tsai F et.al
GSE41177	GPL570	19	16	3	Taiwan	Yeh Y et.al
GSE14975	GPL570	10	5	5	Germany	Adam O et.al

*GSE* Gene Expression Omnibus; *AF* Atrial Fibrillation; *SR* Sinus Rhythm

### Data preprocessing

Series matrix files were processed with ActivePerl 5.24.2 software (https://www.activestate.com/ products/perl/) to convert the gene probe IDs to gene symbol codes. Because GSE14975 was extracted using the Affymetrix Microarray Suite 5 (MAS5) algorithm, it was log base 2 transformed. After merging all microarray data, batch effects were adjusted by the “combat” function of “sva” package of R software using empirical Bayes frameworks [10]. Finally, normalized expression values according to the “normalizeBetweenArrays” function of the package of “limma” in R software so that the expression values have similar distribution across a set of arrays [11].

### Identification of differentially expressed genes (DEGs)

To assess differential expression, using the “limma” package of R software, a linear model was fitted and a simple empirical Bayes model was used to moderate standard errors [11]. A moderated t-statistic and a log-odds of differential expression was computed for each contrast for each gene. The Benjamini and Hochberg (BH) method was performed to adjust *P* value to reduce the false positive error. A gene was defined as a DEG between the AF and SR sample, when the adjust *P* value was < 0.05 and the gene expression fold change (FC) value was ≥1.5 or ≤ 2/3 (|log2 FC| ≥ 0.58), which were visualized as Volcano plots and heat map plots. “ggplot” packages of R software was applied to generate box plots for genes which have the adjusted *P* value < 0.05 and the gene expression FC value ≥2 or ≤ 0.5 (|log2 FC| ≥ 1).

### Functional and pathway enrichment analyses of DEGs

The Gene Ontology (GO) Resource (http://geneontology.org/) is a bioinformatics tool providing a framework and set of concepts for describing the functions of gene products from all organisms [12]. Kyoto Encyclopedia of Genes and Genomes (KEGG) (https://www.kegg.jp/) is a database resource integrated the information of genomes, biological pathways, diseases and chemicals [13]. Gene Set Enrichment Analysis (GSEA) (http://software.broadinstitute.org/gsea/index.jsp) is a computational method for interpreting gene expression data based on molecular signature database [14]. The Reactome Pathway Database (https://reactome.org/) is pathway annotation database collecting the biological pathways and processes in the human [15]. The Disease Ontology (DO) (http://disease-ontology.org) represents a comprehensive knowledge base of 8043 inherited, developmental and acquired human disease [16]. Before performing enrichment analysis, human genome annotation package “org.Hs.eg.db” was used to convert gene symbol codes to Entrez ID. To better understand the biological function and characteristics, R software was used to perform enrichment analysis, with the “clusterProfiler” package for Go and KEGG enrichment analyses, the “ReactomePA” package for Reactome pathway analysis and the “DOSE” package for DO enrichment analysis. The “ggplot2”, “pathview” and “graphite” packages of R software were used to visualize the plots. GO terms and KEGG maps of biological functions associated with an adjusted *P* value < 0.05 and Q value < 0.05 was considered to be significantly enriched. GSEA v4.0.3 was applied for GSEA analysis. Using a permutation test 1000 times, the cutoff of the significance level nominal *P*-value, false discovery rate (FDR) Q-value and family wise-error rate (FWER) *P*-value were all chosen as 0.05 for the most significant pathways related to AF.

### Protein-protein interaction (PPI) network and potential crucial genes analyses

The STRING database (http:/ /string-db.org/) was performed to construct a PPI network to reveal the generic organization principles of functional cell systems and to predict protein-protein interactions [17]. The Molecular Complex Detection (MCODE) of Cytoscape was carried out to module analyze and visualize the result of PPI network. Default parameters (degree cutoff ≥2, node score cutoff ≥2, K-core ≥2, and maxi-mum depth = 100) were used. To select potential crucial genes, we synthesized above significant bioinformatics information for subsequent analyses. DEGs were considered as potential crucial genes if they met the following one of inclusion criteria: (1) Adjust *P* value < 0.05 and |log2FC| ≥ 1; (2) *P* value < 0.05, |log2FC| ≥ 0.58 and enriched in biological functions. In addition, genes with the degree of connectivity larger than 5 in the PPI network were also included.

### Identification of potential crucial genes associated with AF

The comparative toxicogenomics database (CTD, http://ctdbase.org/) integrated information including chemical-gene/protein interactions, chemical-disease and gene-disease relationships to develop hypotheses related to the mechanisms of disease [18]. The association between potential crucial genes and AF risk was analyzed using the data in CTD.

## Results

### Identification of DEGs

**Fig. 1:**
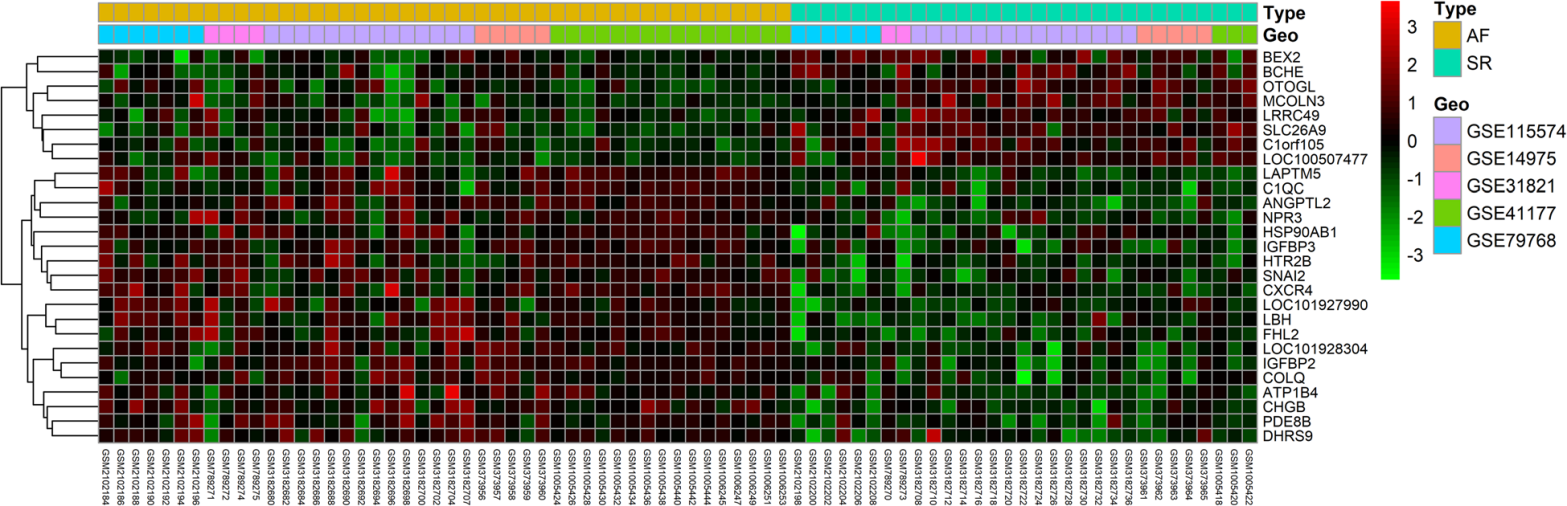
Heat map of DEGs in AF samples and SR samples. Each row represents a sample, and each column represents a single gene. Purple color represented AF samples, and blue color represented SR samples. The color scale shows the relative genes expression level in certain slide: green indicates low relative expression levels; red indicates high relative expression levels

**Fig. 2:**
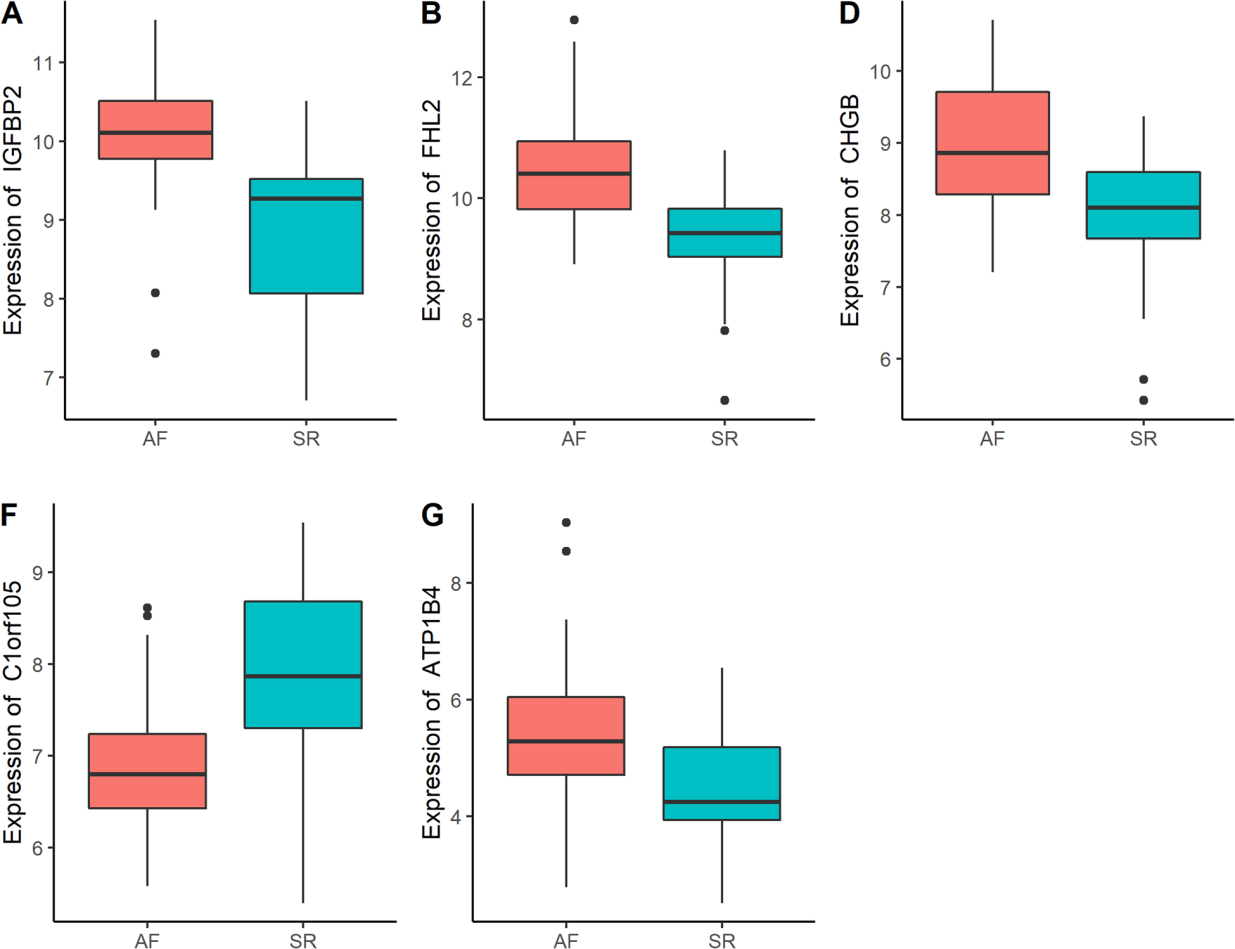
Boxplots of gene expressions for 5 selected genes with a screening criteria of |log2 FC| ≥ 1 and adjust *P* value < 0.05

Gene expression levels of merged GEO series that have been adjusted batch effects were standardized and the results of pre- and post- standardized were presented in Supplementary Figure 1. The 54,675 probes corresponding to 21,654 genes in GSE115574, GSE31821, GSE79768, GSE41177 and GSE14975 datasets were identified and DEGs of AF were confirmed. Twenty-seven of DEGs with |log2 FC| ≥ 0.58 in LAA samples of AF patients compared with SR was identified, including 19 up-regulated genes and 8 down-regulated genes (Supplementary Table 1). Heatmap plot and Volcano plot of 27 DEGs enrolled in subsequent analyses was showed in Fig. 1 and Supplementary Figure 2. Using a screening criteria of |log2 FC| ≥ 1, there were 5 genes identified, with 4 of these genes being up-regulated and 1 down-regulated (Table 2). Boxplots for the 5 selected genes were shown in Fig. 2.

**Table 2:** The DEGs of merged data set with the use of criteria of adjust *P* value < 0.05 and |log2FC| ≥ 1

Gene	Log2FC	AveExpr	t	*P* value	adjust *P* value	B
*IGFBP2*	1.1746	9.5881	−6.0548	< 0.0001	0.0006	8.0172
*C1orf105*	−1.0560	7.2935	5.6106	< 0.0001	0.0011	6.3668
*FHL2*	1.1255	9.9882	−5.4755	< 0.0001	0.0016	5.8742
*CHGB*	1.0836	8.5851	−5.3090	< 0.0001	0.0025	5.2745
*ATP1B4*	1.0469	5.0220	−4.3592	< 0.0001	0.0283	2.0308

*Log2FC* log2 Fold Change; *AveExpr* Average Expression

### Functional enrichment analyses of DEGs

To further investigate the biological functions of the 27 DEGs, functional enrichment analyses were performed and results were shown in Table 3. The result of molecular function in GO revealed that two up-regulated DEGs (*IGFBP2* and *IGFBP3*) were enriched in insulin-like growth factor I binding process (adjusted *P* value = 0.0168 and Q value = 0.0120) and insulin-like growth factor binding process (adjusted *P* value = 0.0432 and Q value = 0.0310). Using screening criteria of adjusted *P* value < 0.05 and Q value < 0.05, no pathway was enriched in KEGG. The ‘mineral absorption’, ‘calcium signaling pathway’ and ‘proximal tubule bicarbonate reclamation’ pathways were enriched (*P* values = 0.0033, 0.0331 and 0.0343 respectively). Specifically, *SLC26A9* and *ATP1B4* genes were enriched in mineral absorption, while two up-regulated genes (*CXCR4* and *HTR2B*) were correlated with calcium signaling pathway. Only up-regulated *ATP1B4* gene was enriched in proximal tubule bicarbonate reclamation pathway. However, these enrichments did not remain significant after multiplicity adjustment by BH.

**Table 3:** Significant enriched GO terms and pathways of DEGs

	Term	Count	Genes	*P* value	adjust *P* value	Q value
GO terms						
GO:0031994	insulin-like growth factor I binding	2	*IGFBP2/IGFBP3*	0.0001	0.0168	0.0121
GO:0005520	insulin-like growth factor binding	2	*IGFBP2/IGFBP3*	0.0006	0.0433	0.0311
KEGG Pathway						
hsa04978	Mineral absorption	2	*SLC26A9/ATP1B4*	0.0033	0.2088	0.2088
hsa04020	Calcium signaling pathway	2	*CXCR4/ HTR2B*	0.0332	0.2741	0.2741
hsa04964	Proximal tubule bicarbonate reclamation	1	*ATP1B4*	0.0343	0.2741	0.2741
Reactome Pathway						
R-HSA-381426	Regulation of Insulin-like Growth Factor (IGF) transport and uptake by Insulin-like Growth Factor Binding Proteins (IGFBPs)	3	*IGFBP2/ IGFBP3/ CHGB*	0.0008	0.0632	0.0435

*GO*Gene Ontology; *DEGs* Differentially Expressed Genes

Pathway enrichment using the REACTOME database identified that three up-regulated DEGs (*IGFBP2*, *IGFBP3* and *CHGB*) were enriched in Regulation of Insulin-like Growth Factor (IGF) transport and uptake by Insulin-like Growth Factor Binding Proteins (IGFBPs) (*P* value = 0.0008 and Q value < 0.0435, Supplementary Figure 3). DO enrichment analysis revealed that DEGs were enriched in 29 biological processes (adjusted *P* value < 0.05 and Q value < 0.05), but not in AF associated process (Supplementary Figure 4).

GSEA was applied to test merged GEO dataset to identify functional gene sets correlated with heart. The SR > AF analysis identified five sets whose expression was correlated with AF (Supplementary Table 2). All were related to heart function (FDR Q-value < 0.05 and FWER *P*-value < 0.05). The sets were (1) regulation of cell growth involved in cardiac muscle cell development, (2) regulation of cardiac muscle cell differentiation, (3) cardiac muscle cell differentiation, (4) positive regulation of cardiac muscle cell differentiation, (5) physiological cardiac muscle hypertrophy. To explore whether these sets contained DEGs, we examined the leading-edge subsets for each gene set (defined above). In five leading-edge subsets, only DEG *FHL2* was involved in cardiac muscle cell differentiation.

### PPI network construction and potential crucial genes selection

Using the STRING platform, PPI analysis of these DEGs identified 18 nodes and 26 interactions. In addition, one significant module with 5 nodes and 9 edges was screened out via MCODE (Supplementary Figure 5a). *CXCR4*, *IGFB P2*, *IGFBP3*, *SNAI2* and *ANGPTL2* were hub nodes in module. Only *CXCR4*, *IGFBP2* and *IGFBP3* were selected for hub genes, all of which were involved in playing pivotal regulatory roles in PPI network, due to the high degree of connectivity (degree ≥5, Supplementary Figure 5b). Furthermore, after combining with the results of differential expression, enrichment analyses and PPI, *IGFBP2*, *IGFBP3*, *CHGB*, *CXCR4*, *HTR2B*, *FHL2*, *C1orf105*, *ATP1B4* and *SLC26A9* were considered potential crucial genes for further analyses.

### Identification of potential crucial genes associated with AF

CTD was employed to explore the interaction between potential crucial genes and AF. As shown in Supplementary Figure 6, potential crucial genes targeting AF, left ventricular dysfunction, heart diseases and cardiovascular diseases. Inference scores in CTD reflected the association between chemical, disease and genes. The interaction results showed that *CXCR4*, *IGFBP2*, *IGFBP3* and *FHL2* have a higher score with AF.

## Discussion

In the present study, we integrated gene expression profiles of 46 AF samples and 31 SR samples from 5 GEO datasets and analyzed the data using bioinformatics tools. A total of 27 DEGs with |log2 FC| ≥ 0.58 and 5 with |log2 FC| ≥ 1 in AF compared with SR samples were selected. Furthermore, 9 potential crucial genes (*IGFBP2*, *IGFBP3*, *CHGB*, *CXCR4*, *HTR2B*, *FHL2*, *C1orf105*, *ATP1B4* and *SLC26A9*), and several important pathways, which were associated with AF risk, were identified, suggesting these may play important role in the mechanism of AF.

*IGFBP2*, located in chromosome 2q35, encodes the second most abundant circulating IGF-binding protein. IGFBP2, secreted by differentiating white adipocytes, regulates the functions of IGFs [19]. In the Framingham heart study, IGFBP2 was significantly associated with all-cause mortality [20]. Our study revealed that the *IGFBP2* expression level was up-regulated in AF samples compared to SR samples. The enrichment analysis results of *IGFBP2* were all correlated with *IGFBP3*.

The functional gene *IGFBP3* encodes the primary carrier of IGFs (IGFBP3) in the circulation. IGFBP3 is involved in oxidative stress, atherosclerosis and left ventricular hypertrophy [21, 22]. More important, a previous study revealed low IGFBP3 serum level as an independent determinant of AF [21]. Enrichment analyses in the present study indicated that *IGFBP2* and *IGFBP3* were enriched in GO term of insulin-like growth factor binding and pathway of IGF transport and uptake by IGFBPs. Thus, IGFBP2 and IGFBP3 might bind and regulate the functions of IGFs through IGF transport and uptake by IGFBPs pathway, and then affect the susceptibility of AF [19].

Our study revealed that *CHGB* was involved in IGF transport and uptake by IGFBPs pathway including *IGFBP2* and *IGFBP3*, and the gene expression of *CHGB* was higher in AF patients. Chromogranin B (CHGB) is an emerging cardiovascular biomarker, which is encoded by *CHGB* [23]. CHGB can regulate B-type natriuretic peptide (BNP) production through polycystin 2(PC2)-CGB-BNP signaling axis in cardiomyocyte, and integrate information from myocardial stress and neuro-endocrine activation [23, 24]. CHGB level was significantly increased in heart failure patients [23]. These findings suggested that IGF transport and uptake by IGFBPs pathway with *IGFBP2*, *IGFBP3* and *CHGB* may participate in the occurrence and development of AF. A more thorough understanding of IGF transport and uptake by IGFBPs pathway in AF is necessary.

With PPI analysis, *IGFBP2*, *IGFBP3* and *CXCR4* were divided into a group according to protein-protein interactions. There was evidence that *CXCR4* was overexpressed in chronic AF patients, and might contribute to the process of AF through regulating atrial fibrosis and structural remodeling [25]. In the present study, *CXCR4* was also found to be potential crucial gene related to AF. KEGG pathway enrichment analysis showed that the *CXCR4* and *HTR2B* were enriched in calcium signaling pathway, which had been extensively characterized in the role in cardiac hypertrophy and remodeling processes [26]. *HTR2B* is located in chromosome 2q37.1. 5-HT_2B_ (5-hydroxytryptamine receptor 2B) receptor coded by *HTR2B*, is presented in the cardiovascular system, and may indirectly produce life-threatening arrhythmias and cardiodepression [27, 28]. In auricular myocytes of newborn rat, the activation of 5-HT_2B_ enhances gap junctional intercellular communication (GJIC) in a receptor subtype-specific manner, and prolongs 5-HT exposure to alter the Cx expression pattern which associated with AF [28].

Increasing evidence demonstrated that *FHL2* and its protein product has a function in cardiovascular disease [29, 30]. FHL2, located at the sarcomere, interacted with extracellular signal regulated kinase (ERK) and regulated cardiac growth, suggesting FHL2 a protective role in adrenergic-mediated cardiac hypertrophy [31, 32]. We found that *FHL2* was up-regulated in AF samples compared to SR, and was involved in cardiac muscle cell differentiation. These results indicated that *FHL2* might be a potential biomarker of AF.

In this study, *C1orf105* and *ATP1B4* had 2 fold lower and higher gene expression, respectively in AF patients than SR control. *SLC26A9* and *ATP1B4* were enriched in KEGG pathways of mineral absorption and proximal tubule bicarbonate reclamation. Previous study showed that a SNP on *C1orf105* was associated with remodeling response to atherosclerosis [33]. *Slc26a9* encodes transporters with diverse functional attributes and RT-PCR showed that Slc26a9 is detectable in heart [34]. The above evidence revealed that *C1orf105*, *SLC26A9* and *ATP1B4* were related with cardiovascular disease and might have a function in AF.

In current study, we have discussed that 9 potential crucial genes are involved in the occurrence and development of AF, suggesting these genes may serve as potential biomarkers and therapeutic targets for AF. However, the limitations of this study should be considered. Firstly, it is difficult to consider some important factors such as regions, races and age. Considering that the development of AF results from various environmental and genetic factors, some unmeasured factors including region, family history and risk factors of AF should be evaluated in further research. In addition, the potential crucial genes need further validation by RT-qPCR in clinical samples. Finally, the mechanisms in which these genes play are not completely clear. More evidence is required to find out the biological foundation.

## Conclusion

Our study integrated data with relative larger sample size from multiple GEO datasets and identified 9 potential crucial genes (*IGFBP2*, *IGFBP3*, *CHGB*, *CXCR4*, *HTR2B*, *FHL2*, *C1orf105*, *ATP1B4* and *SLC26A9*), and pathways using bioinformatic analyses. The exploration of potential crucial genes of AF may provide some potential aid in further identification of new biomarkers for the susceptibility of AF and useful treatment targets.

## Supplementary information

**Additional file 1: Figure S1.** (a) Data standardization. Pre-standardization gene expression levels of each data set are presented as blue boxplots; (b) Data standardization. Post-standardization gene expression levels of each data set are presented as red boxplots.

**Additional file 2: Figure S2.** Volcano plot of DEGs in AF samples compared to SR samples. Red indicates the gene expression was up-regulated in AF samples compared to primary samples (adjust *P* value < 0.05 and |log2 FC| ≥ 0.58); Green indicates the gene expression was down-regulated in AF samples compared with primary samples (adjust P value < 0.05 and |log2 FC| ≥ 0.58); Black indicates the adjusted *P* value was > 0.05.

**Additional file 3: Figure S3.** Pathway view of Regulation of Insulin-like Growth Factor (IGF) transport and uptake by Insulin-like Growth Factor Binding Proteins (IGFBPs) using the REACTOME database. *IGFBP2*, *IGFBP3* and *CHGB* were enriched in the pathway.

**Additional file 4: Figure S4.** Bar plot of DO enrichment of DEGs. The X-axis indicates the number of genes represented in the disease.

**Additional file 5: Figure S5.** (a) PPI network of the DEGs and modular analysis. Yellow nodes represent DEGs in the same module. (b) Bar plot for the interaction numbers of each gene in PPI network.

**Additional file 6: Figure S6.** Relationship to AF diseases related to potential crucial genes based on the CTD database.

**Additional file 7: Table S1.** The DEGs of merged data set with the use of criteria of adjust *P* value < 0.05 and |log2FC| ≥ 0.58. **Table S2.** Summary of GSEA result with FDR Q-value < 0.05 and FWER *P*-value < 0.05.

## Data Availability

Microarray datasets (GSE115574, GSE31821, GSE79768, GSE41177 and GSE14975) for this study are openly available in Gene Expression Omnibus database at https://www.ncbi.nlm.nih.gov/geo/query/acc.cgi?acc=GSE115574, https://www.ncbi.nlm.nih.gov/geo/query/acc.cgi?acc=GSE31821, https://www.ncbi.nlm.nih.gov/geo/query/acc.cgi?acc=GSE79768, https://www.ncbi.nlm.nih.gov/geo/query/acc.cgi?acc=GSE41177 and https://www.ncbi.nlm.nih.gov/geo/query/acc.cgi?acc=GSE14975, respectively (last accessed on 27 Dec 2019).

## Abbreviations

AF: Atrial Fibrillation
DEGs: Differentially Expressed Genes
GEO: Gene Expression Omnibus
FC: Fold Change
LAA: Left Atrial Appendage Tissue
SR: Sinus Rhythm
MAS5: Affymetrix Microarray Suite 5
BH: Benjamini and Hochberg method
GO: Gene Ontology
KEGG: Kyoto Encyclopedia of Genes and Genomes
GSEA: Gene Set Enrichment Analysis
DO: Disease Ontology
PPI: Protein-Protein Interaction
MCODE: Molecular Complex Detection
CTD: Comparative Toxicogenomics Database
IGF: Insulin-like Growth Factor
IGFBPs: Insulin-like Growth Factor Binding Proteins
