# Supplementary figures and images for: Identification of potential crucial genes in atrial fibrillation: a bioinformatic analysis
Source: BMC Med Genomics. 2020 Jul 18;13:104. doi: 10.1186/s12920-020-00754-5 (PMC7368672; doi:10.1186/s12920-020-00754-5)

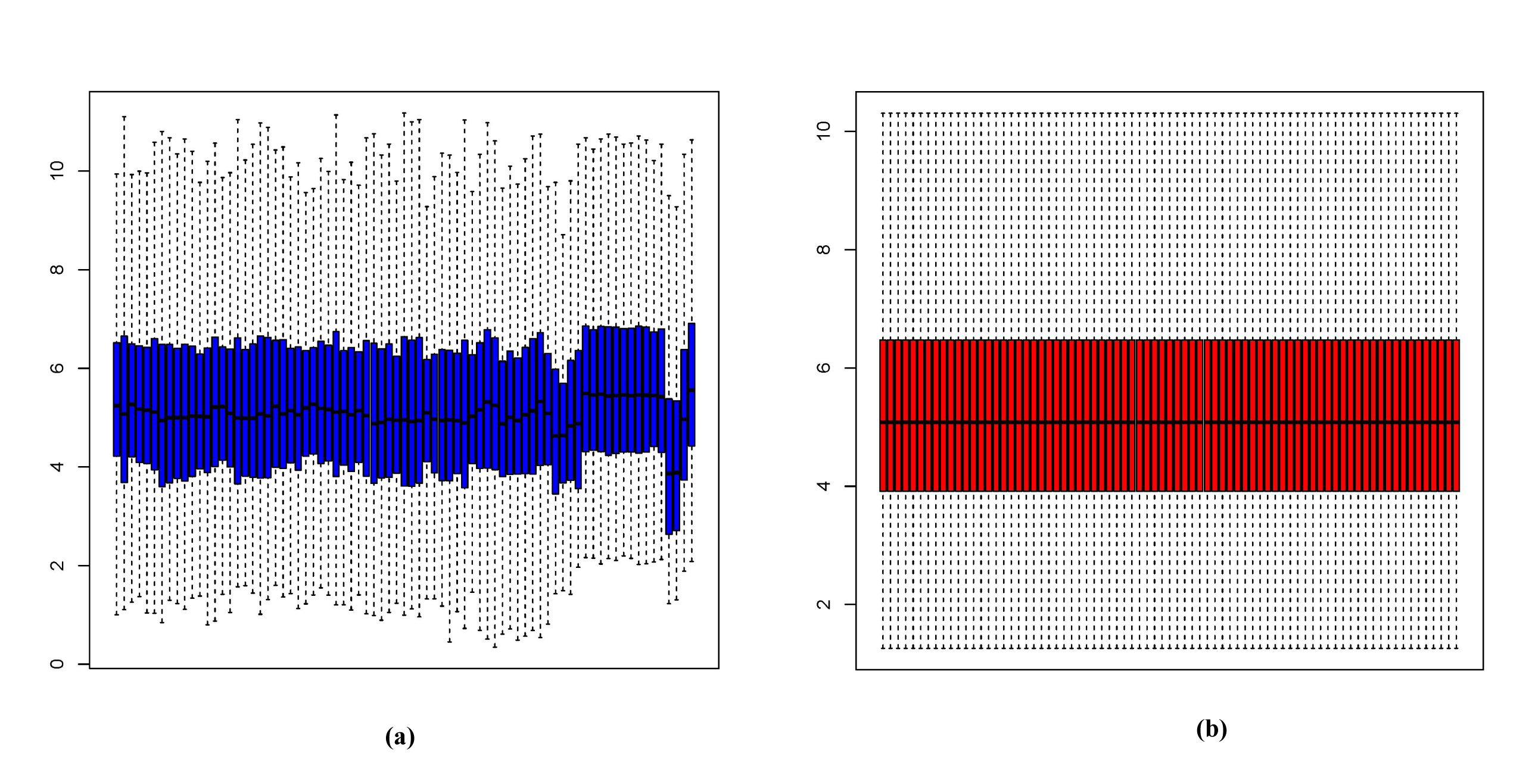

Supplement: Supplementary file 1 — Additional file 1: Figure S1. (a) Data standardization. Pre-standardization gene expression levels of each data set are presented as blue boxplots; (b) Data standardization. Post-standardization gene expression levels of each data set are presented as red boxplots. [file 12920_2020_754_MOESM1_ESM.tif]

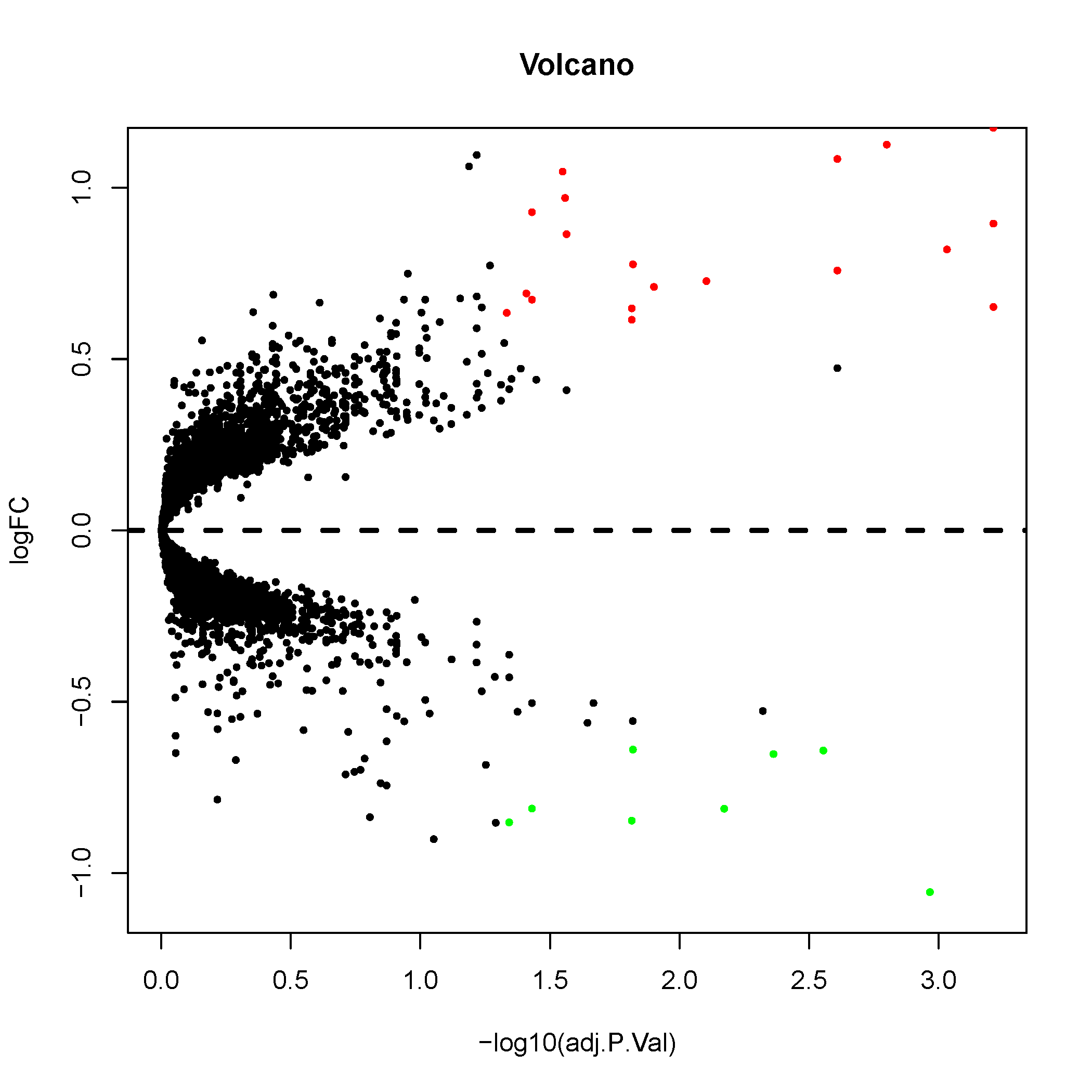

Supplement: Supplementary file 2 — Additional file 2: Figure S2. Volcano plot of DEGs in AF samples compared to SR samples. Red indicates the gene expression was up-regulated in AF samples compared to primary samples (adjust P value < 0.05 and |log2 FC| ≥ 0.58); Green indicates the gene expression was down-regulated in AF samples compared with primary samples (adjust P value < 0.05 and |log2 FC| ≥ 0.58); Black indicates the adjusted P value was > 0.05. [file 12920_2020_754_MOESM2_ESM.tiff]

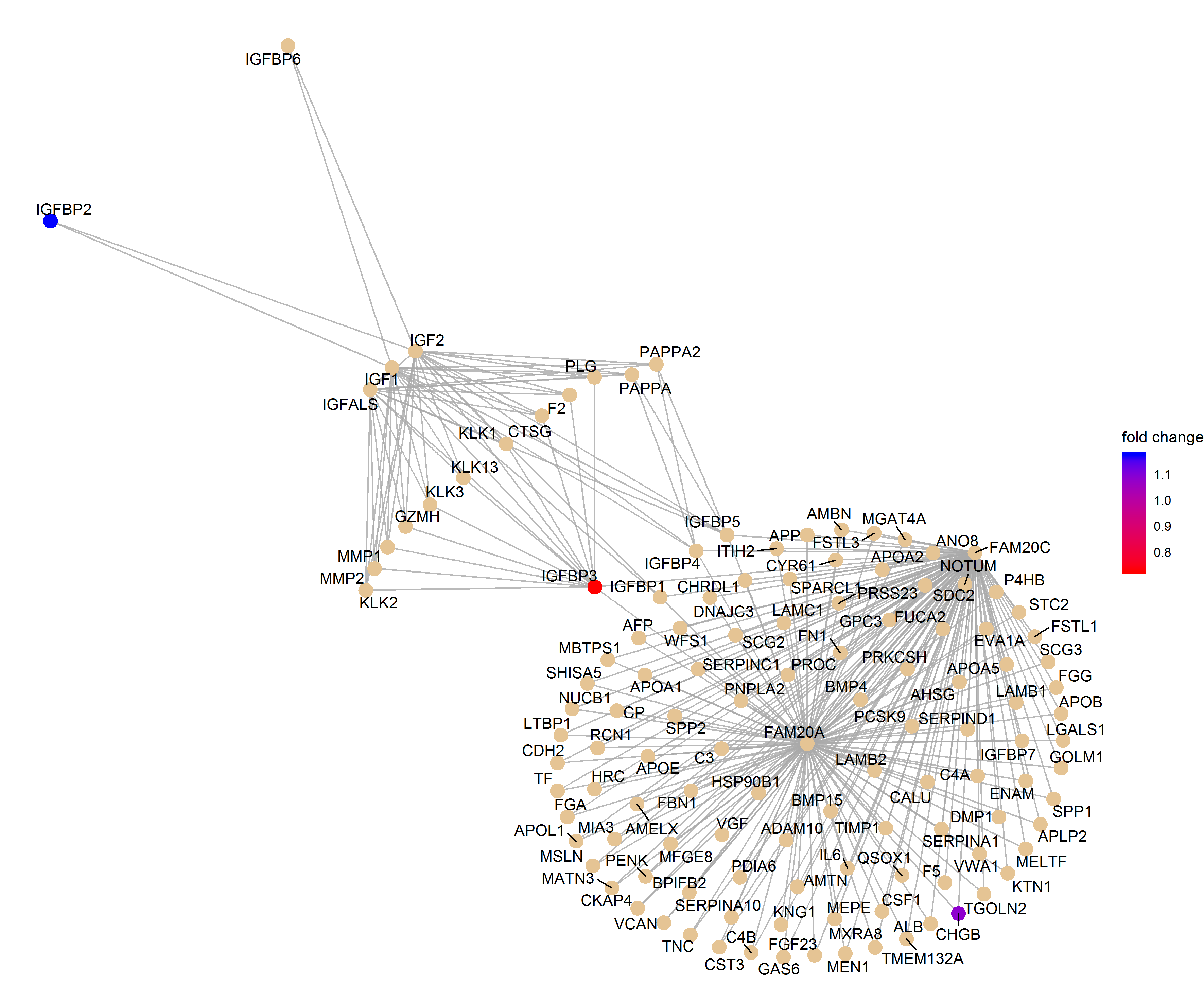

Supplement: Supplementary file 3 — Additional file 3: Figure S3. Pathway view of Regulation of Insulin-like Growth Factor (IGF) transport and uptake by Insulin-like Growth Factor Binding Proteins (IGFBPs) using the REACTOME database. IGFBP2, IGFBP3 and CHGB were enriched in the pathway. [file 12920_2020_754_MOESM3_ESM.tiff]

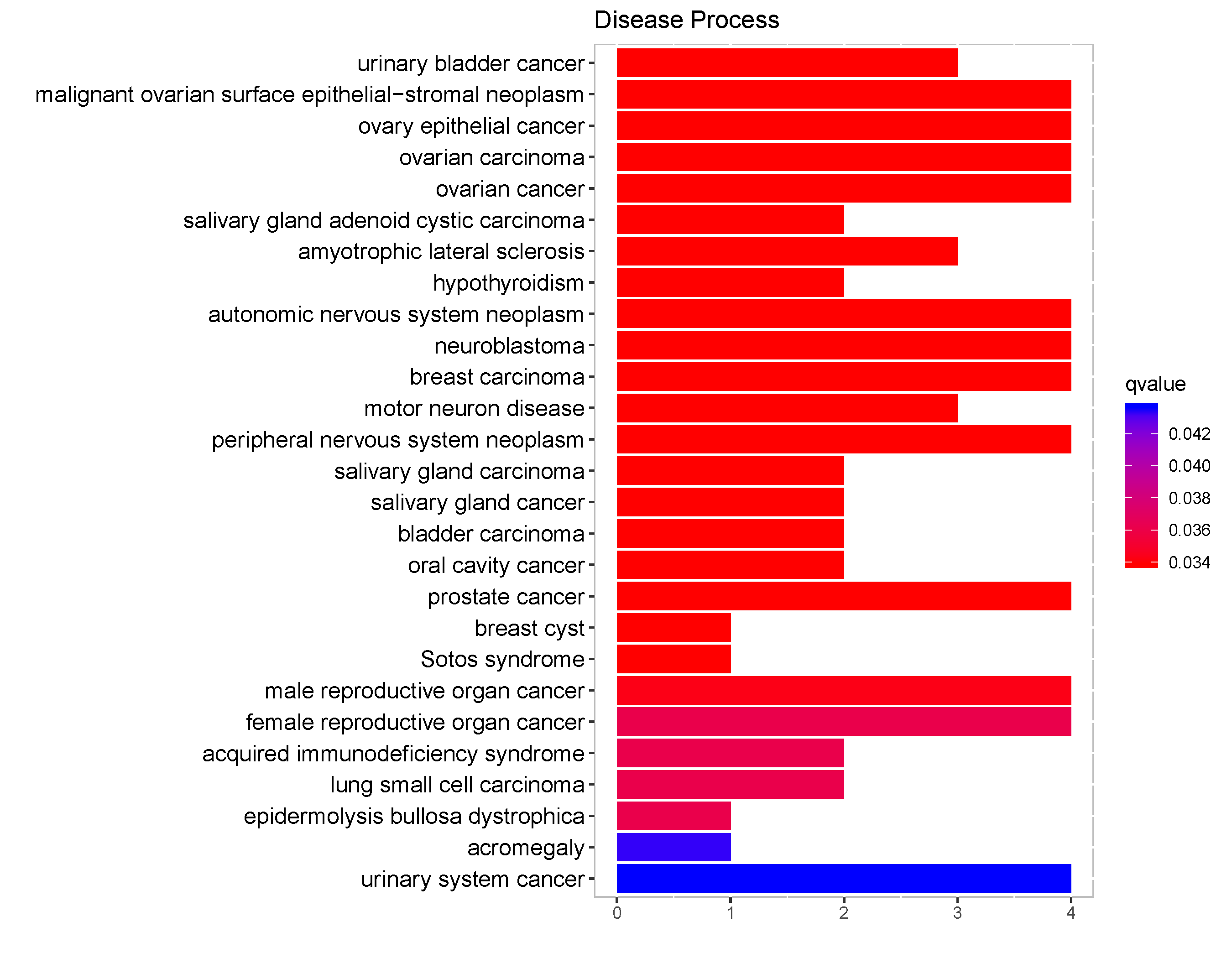

Supplement: Supplementary file 4 — Additional file 4: Figure S4. Bar plot of DO enrichment of DEGs. The X-axis indicates the number of genes represented in the disease. [file 12920_2020_754_MOESM4_ESM.tiff]

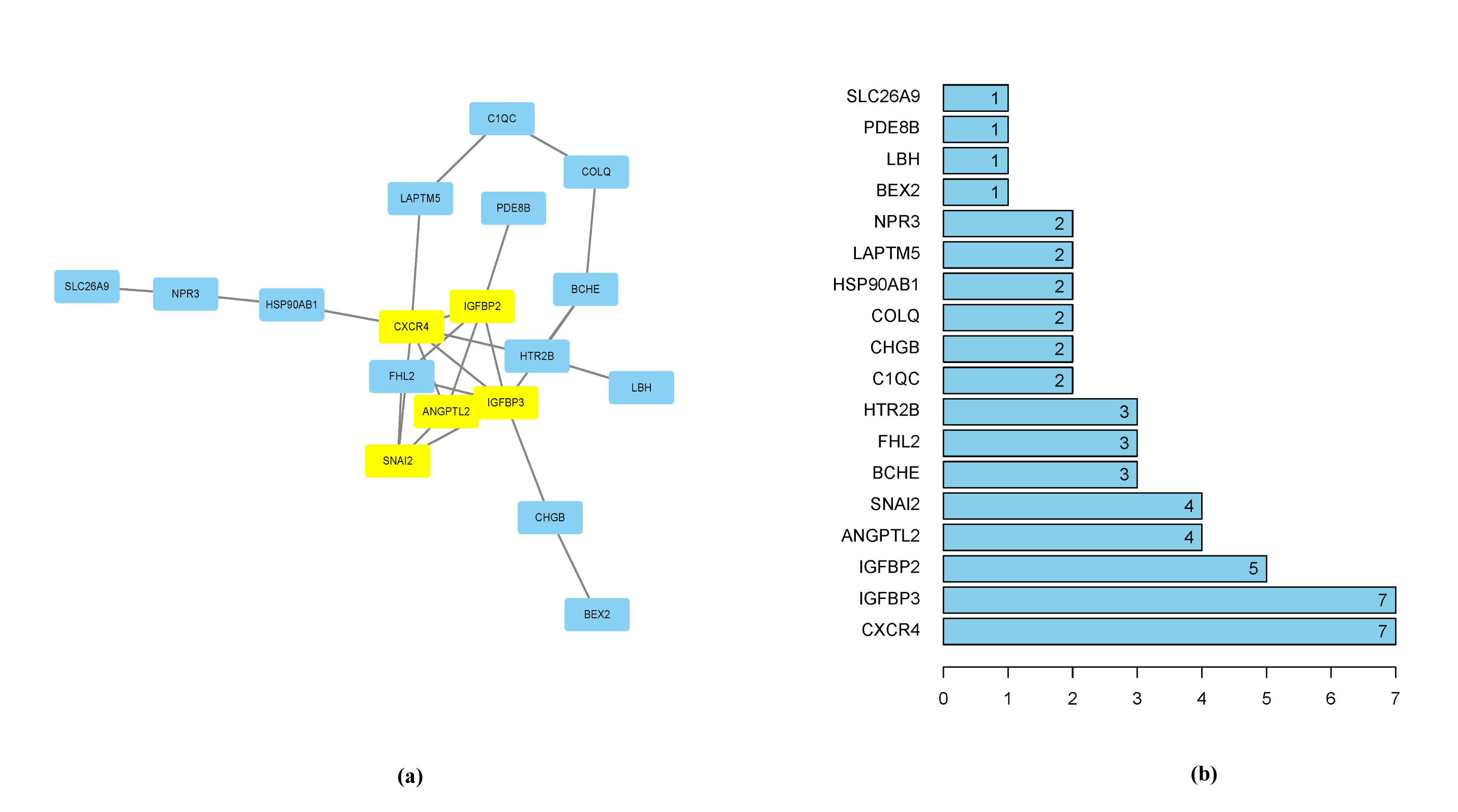

Supplement: Supplementary file 5 — Additional file 5: Figure S5. (a) PPI network of the DEGs and modular analysis. Yellow nodes represent DEGs in the same module. (b) Bar plot for the interaction numbers of each gene in PPI network. [file 12920_2020_754_MOESM5_ESM.tif]

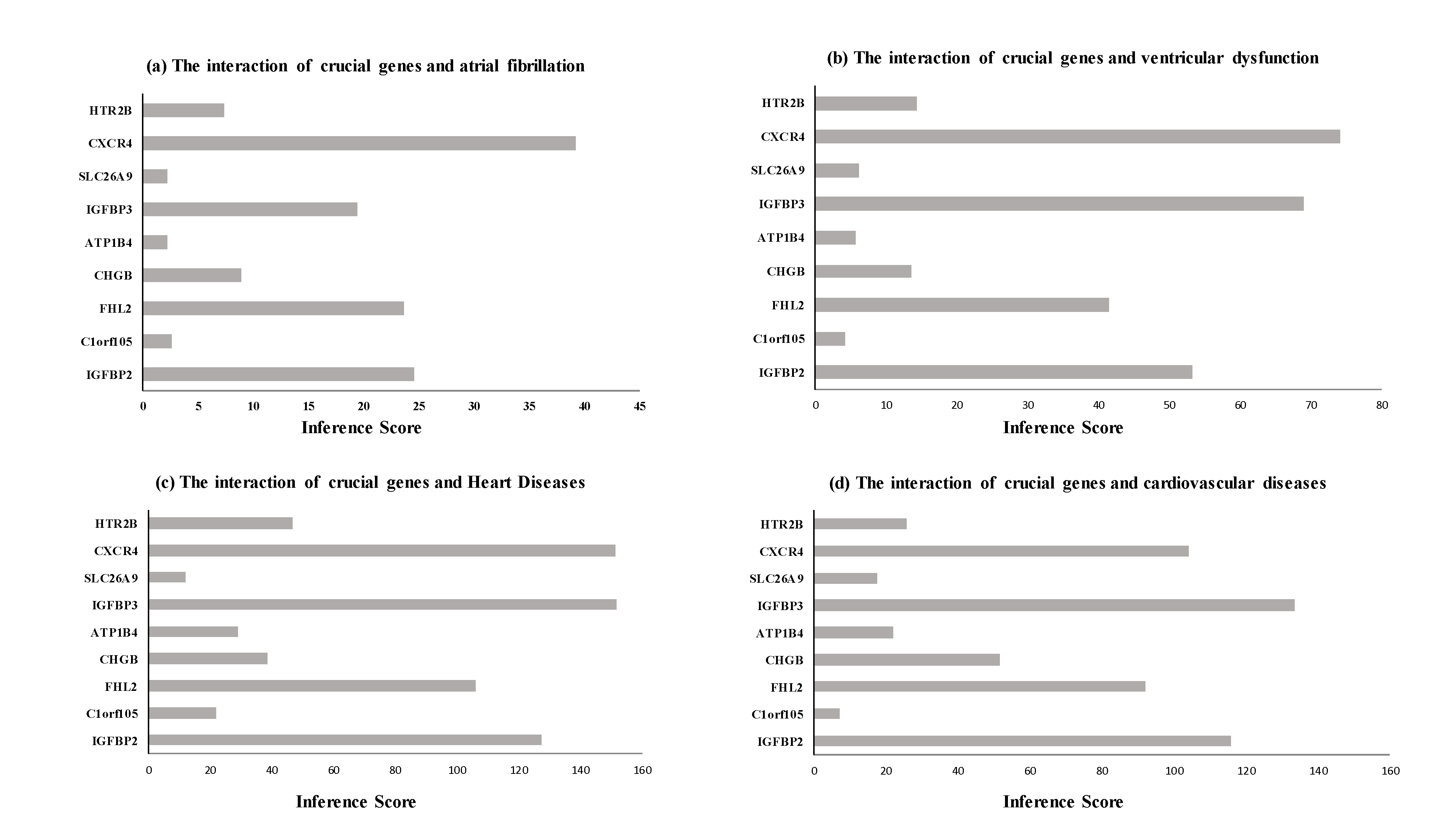

Supplement: Supplementary file 6 — Additional file 6: Figure S6. Relationship to AF diseases related to potential crucial genes based on the CTD database. [file 12920_2020_754_MOESM6_ESM.tiff]
