# Supplementary material for: Identification of potential crucial genes in atrial fibrillation: a bioinformatic analysis
Source: BMC Med Genomics. 2020 Jul 18;13:104. doi: 10.1186/s12920-020-00754-5 (PMC7368672; doi:10.1186/s12920-020-00754-5)
Supplement: Supplementary file 7 — Additional file 7: Table S1. The DEGs of merged data set with the use of criteria of adjust P value < 0.05 and |log2FC| ≥ 0.58. Table S2. Summary of GSEA result with FDR Q-value < 0.05 and FWER P-value < 0.05. [file 12920_2020_754_MOESM7_ESM.docx]

Supplementary Table 1 The DEGs of merged data set with the use of criteria of adjust P value <0.05 and |log2FC|≥0.58

| Gene | logFC | AveExpr | t | P Value | adjust P value | B |
| --- | --- | --- | --- | --- | --- | --- |
| *IGFBP2* | 1.1746 | 9.5881 | -6.0548 | <0.0001 | 0.0006 | 8.0172 |
| *ANGPTL2* | 0.6517 | 7.3149 | -5.9429 | <0.0001 | 0.0006 | 7.5973 |
| *LBH* | 0.8950 | 7.8698 | -5.8640 | <0.0001 | 0.0006 | 7.3026 |
| *SNAI2* | 0.8196 | 7.3771 | -5.6994 | <0.0001 | 0.0009 | 6.6930 |
| *C1orf105* | -1.0560 | 7.2935 | 5.6106 | <0.0001 | 0.0011 | 6.3668 |
| *FHL2* | 1.1255 | 9.9882 | -5.4755 | <0.0001 | 0.0016 | 5.8742 |
| *CHGB* | 1.0836 | 8.5851 | -5.3090 | <0.0001 | 0.0025 | 5.2745 |
| *CXCR4* | 0.7581 | 6.5261 | -5.2774 | <0.0001 | 0.0025 | 5.1613 |
| *SLC26A9* | -0.6428 | 7.6620 | 5.2149 | <0.0001 | 0.0028 | 4.9389 |
| *LOC100507477* | -0.6530 | 6.2490 | 5.0821 | <0.0001 | 0.0043 | 4.4702 |
| *OTOGL* | -0.8126 | 5.1148 | 4.9310 | <0.0001 | 0.0067 | 3.9442 |
| *IGFBP3* | 0.7271 | 9.2341 | -4.8723 | <0.0001 | 0.0079 | 3.7419 |
| *LOC101928304* | 0.7102 | 7.9171 | -4.7358 | <0.0001 | 0.0126 | 3.2767 |
| *MCOLN3* | -0.6401 | 4.9745 | 4.6611 | <0.0001 | 0.0152 | 3.0249 |
| *COLQ* | 0.7761 | 6.8607 | -4.6555 | <0.0001 | 0.0152 | 3.0060 |
| *BEX2* | -0.8475 | 7.9145 | 4.6006 | <0.0001 | 0.0153 | 2.8227 |
| *PDE8B* | 0.6475 | 6.0361 | -4.5990 | <0.0001 | 0.0153 | 2.8172 |
| *LAPTM5* | 0.6144 | 7.4699 | -4.5978 | <0.0001 | 0.0153 | 2.8134 |
| *HTR2B* | 0.8642 | 5.4717 | -4.3992 | <0.0001 | 0.0273 | 2.1602 |
| *LOC101927990* | 0.9699 | 5.5290 | -4.3750 | <0.0001 | 0.0277 | 2.0817 |
| *ATP1B4* | 1.0469 | 5.0220 | -4.3592 | <0.0001 | 0.0283 | 2.0308 |
| *LRRC49* | -0.8121 | 7.9607 | 4.2581 | 0.0001 | 0.0372 | 1.7066 |
| *DHRS9* | 0.9283 | 9.2980 | -4.2458 | 0.0001 | 0.0372 | 1.6675 |
| *NPR3* | 0.6730 | 6.3598 | -4.2412 | 0.0001 | 0.0372 | 1.6528 |
| *C1QC* | 0.6912 | 7.4986 | -4.2194 | 0.0001 | 0.0391 | 1.5837 |
| *BCHE* | -0.8525 | 7.4709 | 4.1370 | 0.0001 | 0.0455 | 1.3243 |
| *HSP90AB1* | 0.6344 | 9.9097 | -4.1133 | 0.0001 | 0.0465 | 1.2505 |

Log2FC, log2 Fold Change; AveExpr, Average Expression

Supplementary Table 2 Summary of GSEA result with FDR Q-value < 0.05 and FWER P-value < 0.05

| Name | Size | ES | NES | NOM  P-value | FDR  Q-value | FWER P-value | Rank at max | Leading edge |
| --- | --- | --- | --- | --- | --- | --- | --- | --- |
| Go Regulation Of Cell Growth Involved In Cardiac Muscle Cell Development | 17 | -0.7928 | -2.3248 | <0.0001 | 0.0428 | 0.004 | 2084 | tags=59%, list=10%, signal=65% |
| Go Regulation Of Cardiac Muscle Cell Differentiation | 34 | -0.7027 | -2.2375 | <0.0001 | 0.0425 | 0.013 | 2084 | tags=50%, list=10%, signal=55% |
| Go Cardiac Muscle Cell Differentiation | 107 | -0.5434 | -2.2250 | <0.0001 | 0.0389 | 0.021 | 3626 | tags=43%, list=17%, signal=51% |
| Go Positive Regulation Of Cardiac Muscle Cell Differentiation | 17 | -0.7894 | -2.1988 | <0.0001 | 0.0423 | 0.031 | 1802 | tags=59%, list=8%, signal=64% |
| Go Physiological Cardiac Muscle Hypertrophy | 26 | -0.6323 | -2.1700 | <0.0001 | 0.0485 | 0.043 | 3331 | tags=50%, list=15%, signal=59% |

ES: Enrichment score; NES: Normalized enrichment score; NOM P-value: Nominal P-value; FDR Q-value: False discovery rate Q-value; FWER P-value: Family wise-error rate P-value
